# Structural and dynamics studies of a truncated variant of CI repressor from bacteriophage TP901-1

**DOI:** 10.1038/srep29574

**Published:** 2016-07-12

**Authors:** Kim Krighaar Rasmussen, Kristian E. H. Frandsen, Elisabetta Boeri Erba, Margit Pedersen, Anders K. Varming, Karin Hammer, Mogens Kilstrup, Peter W. Thulstrup, Martin Blackledge, Malene Ringkjøbing Jensen, Leila Lo Leggio

**Affiliations:** 1 Department of Chemistry, University of Copenhagen, Universitetsparken 5, Copenhagen, Denmark; 2 Univ. Grenoble Alpes, CNRS, CEA, Institut de Biologie Structurale, Grenoble, France; 3 Department of Biology, University of Copenhagen, Ole Maaløes vej 5, DK-2200 Copenhagen N, Denmark; 4 Metabolic signalling and regulation, Department of Systems Biology, Technical University of Denmark, DK-2800 Lyngby, Denmark

## Abstract

The CI repressor from the temperate bacteriophage TP901-1 consists of two folded domains, an N-terminal helix-turn-helix DNA-binding domain (NTD) and a C-terminal oligomerization domain (CTD), which we here suggest to be further divided into CTD_1_ and CTD_2_. Full-length CI is a hexameric protein, whereas a truncated version, CI∆58, forms dimers. We identify the dimerization region of CI∆58 as CTD_1_ and determine its secondary structure to be helical both within the context of CI∆58 and in isolation. To our knowledge this is the first time that a helical dimerization domain has been found in a phage repressor. We also precisely determine the length of the flexible linker connecting the NTD to the CTD. Using electrophoretic mobility shift assays and native mass spectrometry, we show that CI∆58 interacts with the O_L_ operator site as one dimer bound to both half-sites, and with much higher affinity than the isolated NTD domain thus demonstrating cooperativity between the two DNA binding domains. Finally, using small angle X-ray scattering data and state-of-the-art ensemble selection techniques, we delineate the conformational space sampled by CI∆58 in solution, and we discuss the possible role that the dynamics play in CI-repressor function.

Temperate bacteriophages are among several natural bi-stable systems that have the ability to exist within two states of growth. TP901-1 is a temperate bacteriophage of *Lactococcus lactis* that enters either the lytic or the lysogenic lifecycle when infecting a bacterium. In the phage lytic cycle new progeny is synthesized and the bacteria burst, whereas in the lysogenic lifecycle the genome is incorporated into the host genome (prophage state) and the phage is dormant until an external stress factor appears e.g. UV irradiation. UV irradiation initiates DNA damage and the cell’s SOS response, which in turn will make many different prophages enter the lytic life cycle^1^. A genetic switch controls this bi-stability and initiation of lysogenic or lytic gene transcription. In the TP901-1 bacteriophage a genetic switch exist, and is regulated by two divergently oriented promoters P_L_ and P_R_, three operator sites on the DNA (O_D_, distant located operator; O_L_, left operator; O_R_, right operator) and two proteins, the Modulator of Repression (MOR) and the repressor, CI (known as Clear I in λ phage)^2^,^3^,^4^.

CI repressors from phages consist of an N-terminal helix-turn-helix (HTH) domain (NTD) and a C-terminal oligomerization domain (CTD) connected by a linker. Several attempts to solve the three-dimensional structures of CI repressor proteins from various bacteriophages have been made, however, their inherent flexibility has hampered studies of the full-length proteins at atomic resolution. Important insight has, however, been obtained from studies of individual domains and truncated versions of the CI proteins^5^,^6^,^7^,^8^. As an example, the structure of the full-length λ phage CI in complex with one of its operator sites was solved^9^ 21 years after the first crystal structure of its DNA binding domain, highlighting the technical challenges associated with structural studies of such flexible multi-domain proteins. Although their biological function is essentially the same, namely repression, the molecular mechanisms and arrangements needed to modulate repression and cooperativity between operator sites can be fundamentally different between CI proteins from different phages. For example, two of the most studied bacteriophages, λ phage and phage 186, have been found to form either an octamer or a dimer of heptamers upon repression, respectively^6^,^7^,^8^.

CI from TP901-1 consists of an NTD responsible for DNA binding and a CTD responsible for oligomerization^10^,^11^. The crystal structure of NTD has been determined^12^, while the structure of CTD is unknown. The full-length protein has been shown to be a hexamer in solution, where CTD is responsible for the oligomerization state of the protein^10^. A model of repression by CI in TP901-1 has been proposed, where the protein forms a hexameric wheel around which the DNA loops^10^. Thus the molecular mechanism of CI from TP901-1 is fundamentally different from other described phages. MOR, the antirepressor encoded by the *mor* gene, is believed to interact with CI, derepressing P_L_ – from which the lytic genes are transcribed - while allowing the complex to repress P_R_ – from which CI is transcribed. Several aspects of the molecular mechanisms of the TP901-1 switch, which clearly differ from other phage switches, remain poorly understood.

Linkers can play crucial roles in the function of phage CI proteins. A linkerless mutant of the λ phage CI was not able to bind DNA cooperatively, most likely due to the loss of range of distance over which the C-terminal domains can interact with each other^13^. Mutational studies of TP901-1 CI, introducing five additional residues at position Pro78 and at position Ser89 of CI, after the NTD, have demonstrated that this region is important for maintaining bi-stability^11^. The studies show that the mutated CI repressor still has the ability to repress the two divergently oriented promoters P_L_ and P_R_, however only in the absence of the functional *mor* gene. If functional *mor* is present the system loses its bi-stability as P_L_ is always found open^11^ suggesting that the length of the region between NTD and CTD in the CI repressor from TP901-1 plays a crucial role for the bi-stability of the switch.

In this study, we employ a combination of biophysical and biochemical techniques to study the structure, conformational dynamics and DNA-binding of a truncated dimeric version, CI∆58, of CI from TP901-1. CI∆58 and other truncated versions have previously been the subjects of a number of *in vitro* and *in vivo* studies aimed at understanding the TP901-1 switch, and a better understanding of their molecular features is necessary to further interpret these biochemical and genetic studies^4^,^10^,^14^,^15^. Here we combine information from nuclear magnetic resonance (NMR) spectroscopy and a crystal structure to obtain the precise location and length of the linker connecting NTD and CTD. From NMR we also obtain the secondary structure of CI∆58 including the dimerization region that we also analyse in isolation by SEC and CD spectroscopy. Finally, using small angle X-ray scattering data and state-of-the-art ensemble selection techniques, we delineate the conformational space sampled by CI∆58 in solution, and we discuss the possible role that the dynamics play in CI-repressor function.

## Results

### CI∆58 is a modular protein with helical regions of differential flexibility

**Figure 1:**
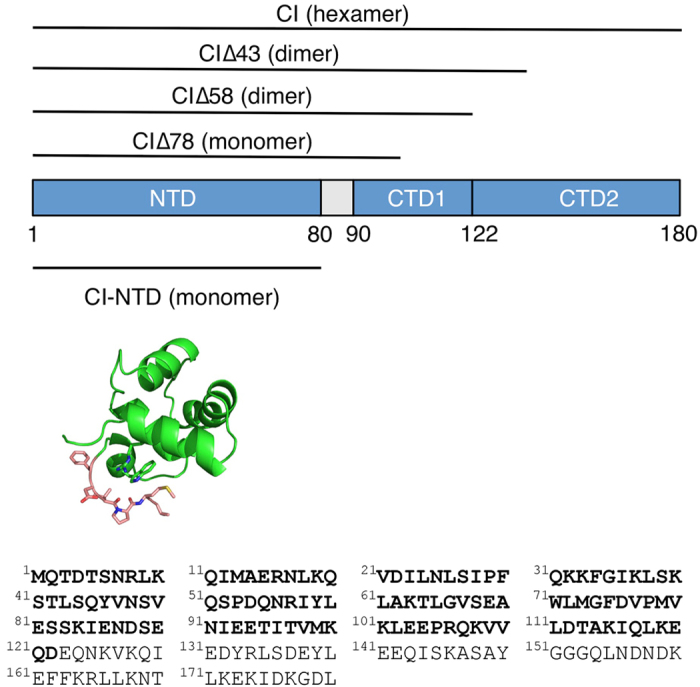
Domain organization of the CI repressor from TP901-1. The oligomerization states of different constructs are indicated. The structure of CI-NTD determined here by X-ray crystallography contains six additional residues (in salmon) compared to the previously determined structure. The sequence of CI (1–180) is shown, and CI∆58 (1–122) is highlighted in bold.

**Figure 2:**
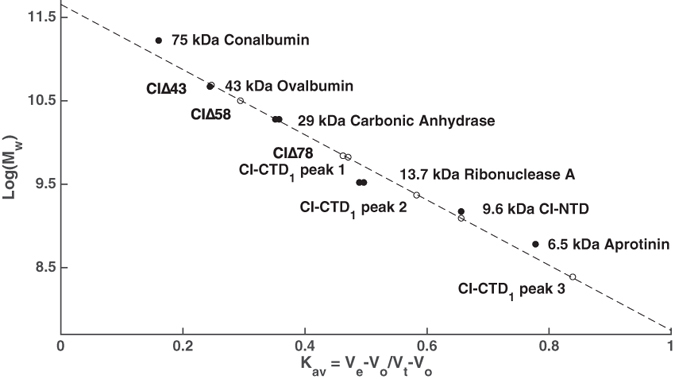
Estimating M_w_ from size exclusion chromatography. All truncated variants and synthesized peptide (CI∆43, CI∆58, CI∆78, CI-NTD and CI-CTD_1_) were gel filtrated on a Superdex 75 Prepgrad 26/600 total volume (Vt) 320 mL. Globular protein standards (closed circles) Conalbumin (75 kDa), Ovalbumin (43 kDa), Carbonic Anhydrase (29 kDa), Ribonuclease A (13.7 kDa), CI-NTD (9.6 kDa) and Aprotinin (6.5 kDa) were used to create a standard curve for estimating molecular weight of unknown samples (open circles) using linear regression equation Log(M_w_) = −3.8875*K_av_+11.6524. Since Conalbumin and Aprotinin seem to deviate from linearity, standard curves were also constructed with one or both omitted. Estimated oligomeric states for all constructs from all standard curves are shown in Table S1.

We expressed and purified CI∆58 that encompasses residues 1–122 of CI TP901-1 (Fig. 1). Size exclusion chromatography (SEC) of CI∆58 unequivocally shows a dimeric protein (Fig. 2, Table S1), as also reported previously^10^. For comparison, CI∆43 is eluted corresponding to a dimer or possibly a trimer, as previously reported, while CI∆78 elutes as a monomer (Fig. 2, Table S1).

**Figure 3:**
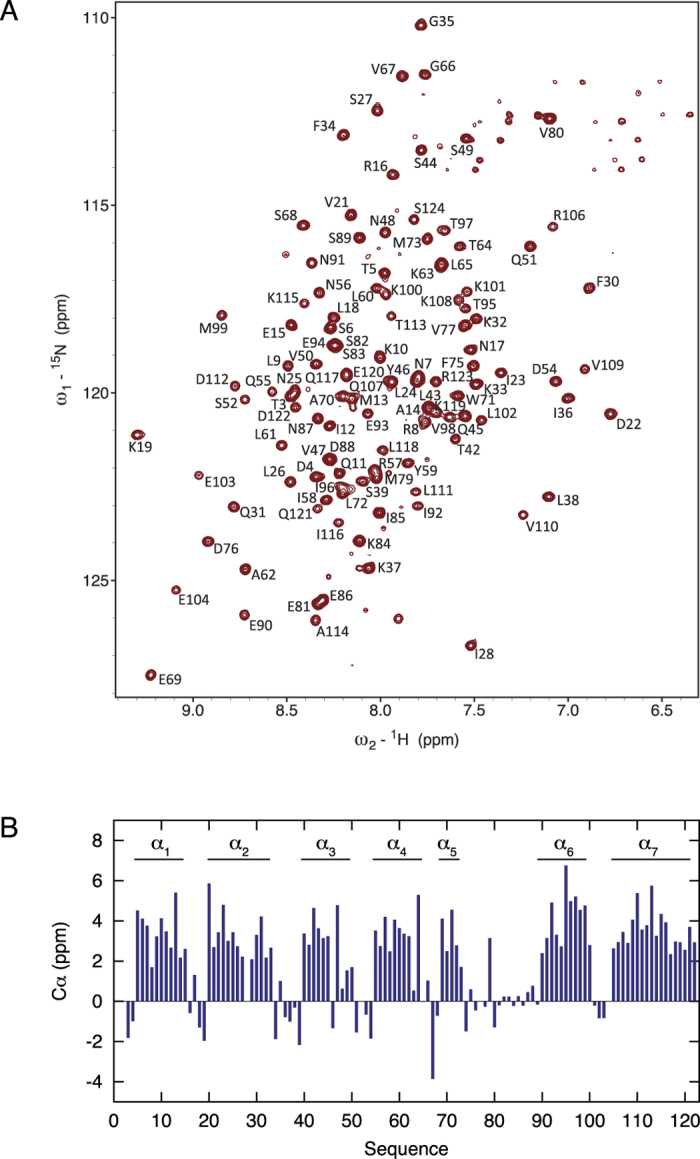
Secondary structure of CIΔ58 obtained by NMR. (**A**) Assigned ^1^H-^15^N TROSY HSQC spectrum of CIΔ58 obtained at a ^1^H frequency of 600 MHz and 311 K. (**B**) Secondary Cα chemical shifts identifying seven α-helices in CIΔ58.

Circular dichroism (CD) of CI∆58 was measured at a wavelength of 222 nm as a function of temperature clearly displaying a three-state unfolding of CI∆58, with two melting temperatures, T_m1_ = 42.7 °C and T_m2_ = 64.2 °C. For comparison, thermal unfolding of the monomeric CI-NTD construct (Fig. S1) resulted in a two-state unfolding with a T_m_ = 58.1 °C (Fig. S1). These observations suggest that CI∆58 is a modular protein consisting of two separate domains. It is unlikely that the observed T_m_s represent transitions from dimer to monomer, since the CD signal at 222 nm depends mostly on secondary structure, rather than tertiary association. To obtain insight into the secondary structure and dynamics of CI∆58, we carried out solution NMR studies. The ^1^H-^15^N TROSY NMR spectrum of CI∆58 displays a single set of resonances (Fig. 3A) demonstrating that the protein forms a symmetric dimer in solution. We carried out the backbone assignment of the N, H^N^, C′ and Cα nuclei of CI∆58 (Fig. 3A, BMRB ID: 26058) and the secondary chemical shifts show that CI∆58 is composed of seven α-helices (Fig. 3B), of which the first five are in agreement with previous crystallographic and NMR studies of CI-NTD^12^. The data additionally show that the latter region of CI∆58 is composed of two α-helices, α_6_ and α_7_, connected by a short loop (Fig. 3B).

**Figure 4:**
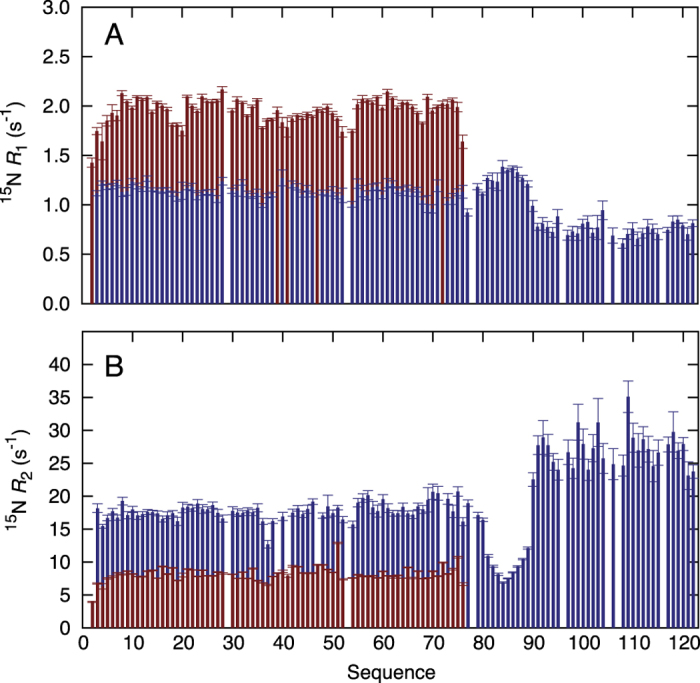
Dynamics of NTD and CIΔ58 from ^15^N nuclear spin relaxation. (**A**) ^15^N *R*_1_ relaxation rates. (**B**) ^15^N *R*_2_ relaxation rates. In all panels, data for NTD and CIΔ58 are shown in red and blue, respectively. All relaxation rates were obtained at a ^15^N frequency of 60 MHz and 298 K.

The ^15^N *R*_1_ and *R*_2_ relaxation rates of CI∆58 show that the protein consists of three regions of differential dynamics corresponding to CI-NTD, a linker region and the additional helical region, hereon referred to as CI-CTD_1_ (Figs 1 and 4). Importantly, we observe high *R*_2_ and low *R*_1_ relaxation rates in CI-CTD_1_ compared to the remainder of the chain, thereby identifying this domain as the dimerization region, in agreement with the three-state thermal denaturation studies. We obtained an average correlation time from the *R*_2_/*R*_1_ ratios for the different regions of CI∆58: 12.1 ns (CI-NTD, residues 2–80), 7.4 ns (linker, residues 81–89) and 18.3 ns (CI-CTD_1_, residues 90–122). Comparing the relaxation data of CI∆58 with those of the isolated CI-NTD (residues 2–74), a large increase in the rotational correlation time of CI-NTD is observed (5.5 ns versus 12.1 ns) in agreement with the increased protein size and the fact that NTD experiences a restriction of motion in the context of the full CI∆58 assembly (Fig. 4).

### Extended crystal structure of NTD

Extensive crystallization efforts were made for CI∆58, but no crystals were obtained. Co-crystallization experiments with DNA were also carried out both with CI∆58 and CI∆43 (Fig. 1). Unfortunately almost all crystals diffracted to worse than 20 Å, which is most likely due to protein flexibility resulting in poor crystal packing. One of the crystals diffracted significantly better, however turned out to contain only protein (DNA was absent). Furthermore residues beyond Val80 were not visible suggesting that the better diffracting crystals were obtained because of limited proteolysis occurring during the long crystallization period.

The resulting crystal structure is essentially the CI-NTD domain, well described in^12^, but contains six additional C-terminal residues compared to the previous structure, and is hereon denoted NTD_80_ (Fig. 1). The six additional residues mainly consist of hydrophobic amino acids and interact closely with the rest of the domain. The extended region is stabilized by hydrogen bonds of the Phe75 backbone carbonyl oxygen with the side chain of Arg8 and the backbone amide of Val77 as well as Met79 π-interactions with Trp71 and a hydrogen bond between the carbonyl group of Met79 and the backbone N of Ser68 (Fig. S2, see Table S2 for statistics).

### Interaction between CI∆58 and DNA

To investigate the behavior of the flexible linker upon DNA interaction, a titration of CI∆58 with the O_L_ site was carried out. As observed in previous studies with CI-NTD^12^ the resonances corresponding to residues in CI-NTD undergo chemical shift and intensity changes in accordance with a DNA interaction occurring on the fast to intermediate chemical shift time scale. No chemical shift changes were observed for residues in the dimerization or linker regions indicating that only CI-NTD interacts directly with the DNA. Approaching the final titration point, also the resonances of the dimerization region of CI∆58 disappear probably due to the overall size of the complex (41.6 kDa). Interestingly the only peaks still present at the end of the titration correspond to residues of the linker region (Fig. S3), showing that it maintains some flexibility upon interaction with DNA.

**Figure 5:**
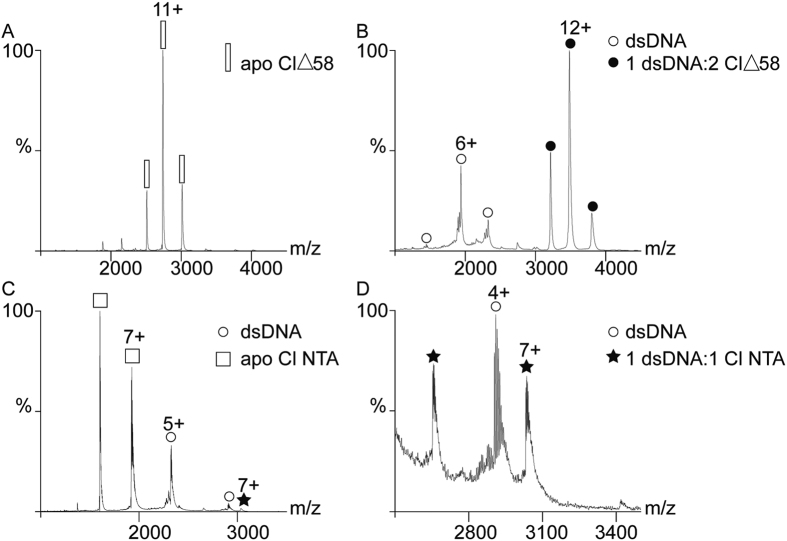
Native mass spectrometry of non-covalent assemblies. (**A**) Apo form of CI∆58. (**B**) CI∆58 incubated with dsDNA. In addition to the non-covalent complex, an unbound dsDNA form was also present. (**C**) CI-NTD incubated with dsDNA, whose spectrum was recorded in the 1000–3500 *m/z* range. The apo CI-NTD and unbound dsDNA represent the strongest signals. (**D**) CI-NTD mixed with dsDNA and analysed in the 2500–3500 *m/z* range. The CI-NTD dsDNA complex was clearly observed. White rectangle = apo form of CI∆58; white sphere = dsDNA; black sphere = CI∆58 dsDNA complex; white square = apo form of CI-NTD; black star = CI-NTD dsDNA complex.

**Figure 6:**
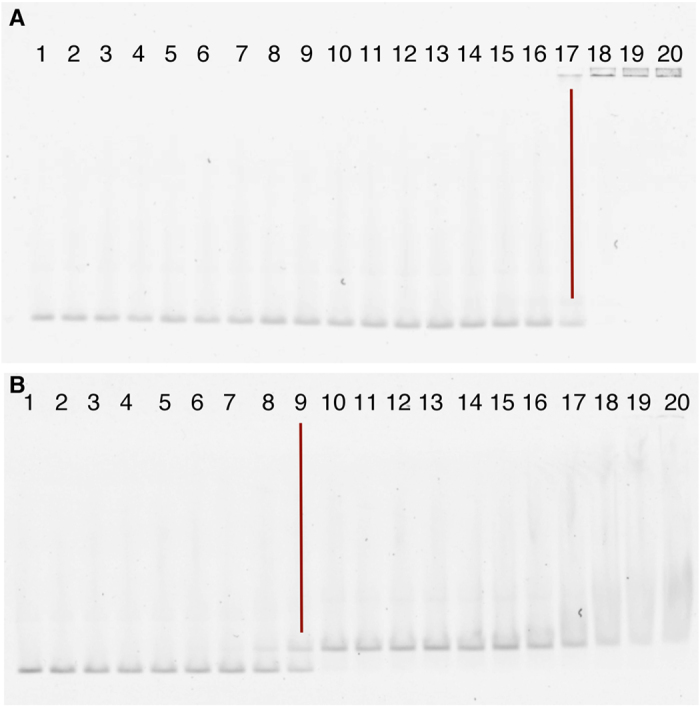
Gel retardation. (**A**) Gel retardation of CI-NTD on fluorescent DNA fragment P5 (containing O_L_) (0.4 nM). K_d_ lies around 1100 nM: lane 1, 0.016 nM; 2, 0.032 nM; 3, 0.065 nM; 4, 0.13 nM; 5, 0.26 nM; 6, 0.52 nM; 7, 1.0 nM; 8, 2.1 nM; 9, 4.1 nM; 10, 8.3 nM; 11, 17 nM; 12, 33 nM; 13, 66 nM; 14, 130 nM; 15, 270 nM; 16, 530 nM; 17, 1100 nM; 18, 2100 nM; 19, 4300 nM; 20, 8500 nM. (**B**) Gel retardation CI∆58 on fluorescent DNA fragment P5 (containing O_L_) (0.4 nM). K_d_ lies around 3 nM: lane 1, 0.011 nM; 2, 0.023 nM; 3, 0.045 nM; 4, 0.090 nM; 5, 0.18 nM; 6, 0.36 nM; 7, 0.72 nM; 8, 1.4 nM; 9, 2.9 nM; 10, 5.8 nM; 11, 12 nM; 12, 23 nM; 13, 46 nM; 14, 92 nM; 15, 180 nM; 16, 370 nM; 17, 740 nM; 18, 1500 nM; 19, 3000 nM; 20, 5900 nM. Red line indicates K_d_.

In order to obtain insight into the stoichiometry of DNA binding, we studied the interaction between CI∆58 and the dsDNA corresponding to the O_L_ operator site by native mass spectrometry (MS). Apo CI∆58 presents a mass of 30107 ± 2 Da, corresponding to the expected dimeric form (Fig. 5A). After incubation with dsDNA (Fig. 5B), CI∆58 formed a non-covalent complex with a mass of 41714 ± 2 Da, corresponding to a dimer of CI∆58 and one dsDNA fragment. The unbound form of dsDNA is also present in the spectrum, having a mass of 11604 ± 3 Da. In contrast, the monomeric CI-NTD binds to the dsDNA with low affinity (Fig. 5C), generating a complex with a mass of 21209 ± 2 Da, corresponding to one dsDNA oligonucleotide and a monomeric CI-NTD. In the *m/z* range 1000–3500, the free dsDNA and CI-NTD (mass: 9607 ± 1 Da) were main signals. When the spectrum was recorded in the 2500–3500 *m/z* range, the CI-NTD dsDNA complex was clearly detectable (Fig. 5D). Dissociation constants for the interaction between CI∆58 or CI-NTD with dsDNA were obtained from Electrophoretic Mobility Shift Assays (EMSA) and were estimated to ~3 nM and ~1100 nM, respectively (Fig. 6A,B). Combined, these results show that in the dimeric construct the NTD domains bind cooperatively to DNA.

### Characterization of CI-CTD_1_

In order to characterize the dimerization region, a synthetic peptide corresponding to residues 90–125 (CI-CTD_1_) was investigated by SEC, CD and SAXS. Initially, problems were encountered with solubility, but a protocol for solubilization was established (see Methods). SEC analysis showed a major peak of higher M_w_ and two minor peaks corresponding to lower M_w_ (Fig. 2, Table S1 and Fig. S4). One of the (very minor) lower M_w_ peaks is clearly the monomer, while the M_w_ of the two other peaks could correspond to a tetramer (major peak) and a trimer or dimer (minor peak) (Table S1, Fig. S4 and Methods section). CD analysis performed on the DichroWeb server^16^ using CDSSTR reference set 1, 2 and 5^17^ and CD spectrum of CTD_1_ (Fig. S5) showed primarily α-helical content, consistent with the NMR analysis of the same region in CI∆58. Furthermore the signal at 208 nm, indicates the presence of interacting α-helices, which can also be inferred from the [Ө]_222_/[Ө]_208_ ratio calculated to 0.911 (Fig. S5)^18^. SAXS analysis was also attempted, but despite the new formulation, it was hampered by aggregation. Thermal unfolding studies with CD were carried out as for CI-NTD and CI∆58 showing a T_m_ of 39.3 °C (Fig. S1) coinciding with one of the observed unfolding transitions of CI∆58.

### Small angle X-ray scattering (SAXS) analysis of CI∆58

To obtain information about the overall shape and dimensions of CI∆58 we used SAXS. The scattering intensity approaches the forward scatter without indications of aggregation and is linear in the Guinier region (Fig. S6A,B). The molecular weight was estimated from the Guinier approximation and cross-validated by the Porod volume V_p_ (Table S3). Interestingly the radius of gyration, *R*_g_, of CI∆58 is similar to BSA although BSA is approximately two times larger in volume. This indicates that CI∆58 is less compact than BSA probably due to the presence of the flexible linker. The Kratky plot^19^ of CI∆58 (Fig. S6C) shows two maxima at s = 0.75 and 1.46 nm^−1^ and a tail that increases with increasing s. These features are in agreement with the presence of multiple (and potentially partially folded) domains consistent with the extracted *R*_g_ values and the NMR relaxation rates.

**Figure 7:**
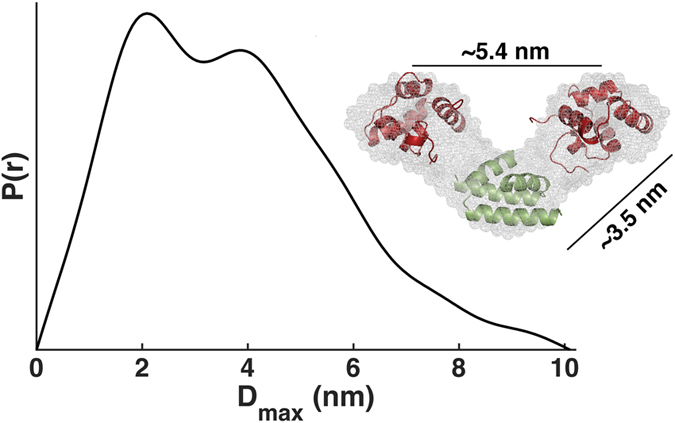
Analysis of experimental SAXS data of CIΔ58. Pair distribution function of CIΔ58 derived from the experimental scattering curve. Insert shows NTDs (red) and model of the dimerization region (green) manually docked into the reconstructed *ab initio* envelope of CI∆58.

*Ab initio* models were reconstructed from the pair distribution function P(r) (Fig. 7). The weighted means and normalized special discrepancies for reconstruction using either P1 or P2 symmetry do not differ significantly (Table S4) reinforcing the evidence for a symmetrical dimer. The *ab initio* model shows that the two CI-NTDs of CI∆58 are on average separated by approximately 5.4 nm, while the average distance between either one of the two NTDs and the dimerization region is approximately 3.5 nm (Fig. 7). The crystal structure of the CI-NTD fits well within the envelope in terms of dimensions.

**Figure 8:**
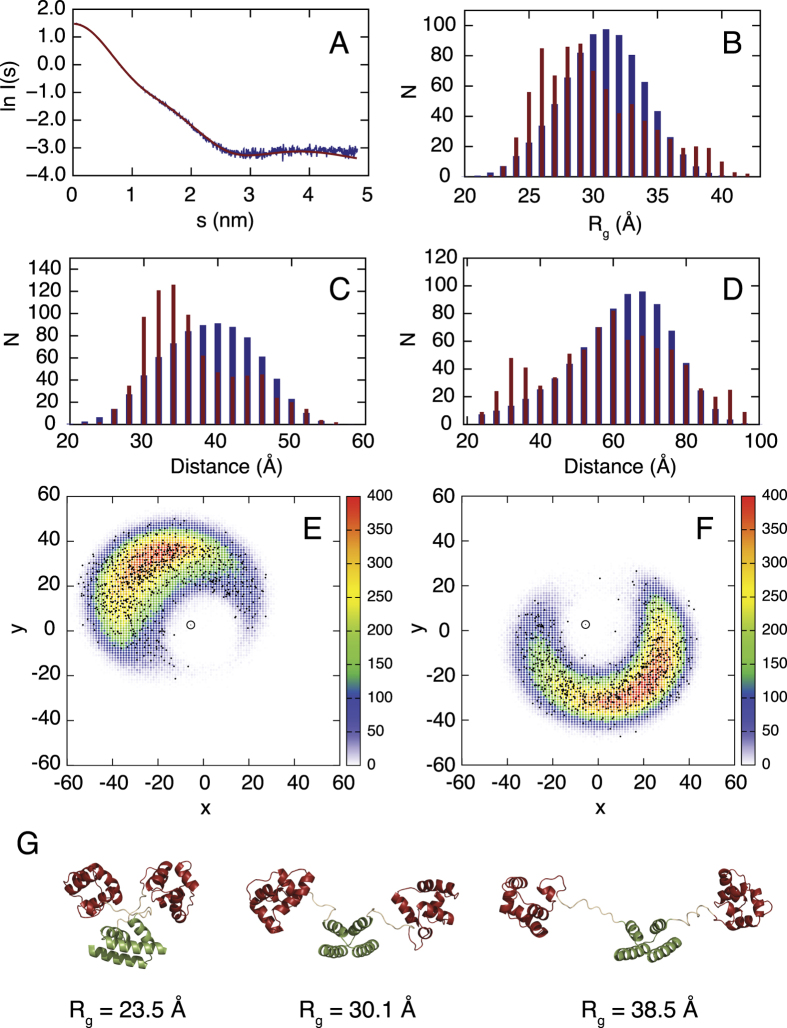
Analysis of experimental SAXS data of CIΔ58 in terms of a structural ensemble description. (**A**) Experimental SAXS curve (blue) and back-calculated SAXS curve from one of the selected ensembles comprising 20 structures (red). (**B**) Distribution of the radius of gyration (*R*_g_) of the pool comprising 400.000 conformers of CIΔ58 (blue) and the distribution of *R*_g_ in the selected ensembles (40 ensembles of 20 structures each, red). (**C**) Distribution of the distance between NTD and the dimerization region in the pool (blue) and in the selected ensembles (red). Similar profiles are obtained for the two NTD domains. (**D**) Distribution of the distances between the two NTDs in the pool of CIΔ58 (blue) and in the selected ensembles (red). The distances were calculated between the centre of mass of the two domains obtained using the coordinates of the Cα atoms. (**E**) Distribution of the centre of mass of a single NTD in the pool projected onto the *xy* plane (colours). The black open circle indicates the centre of mass of the dimerization region. The black points indicate the position of the selected 800 conformers using ASTEROIDS. (**F**) As in (**E**) but for the second NTD. (**G**) Selected conformers representing the two extremes *R*_*g*_ (23.5 Å and 38.5 Å) and the mean *R*_*g*_ 30.1 Å.

The reconstructed static SAXS model presented above does not correctly account for the flexibility of CI∆58 due to the presence of the linker region. We therefore extended our SAXS analysis by interpreting the data in terms of a structural ensemble. In order to do so, a model of the dimerization region had to be created (see Supplementary Note, Table S5 and Fig. S7). We chose to use a two-helical hook model as obtained by the PHYRE2 prediction server (Table S5, Fig. S7) which is consistent with the secondary structure obtained from the NMR data, bioinformatics predictions as well as the *ab initio* model of CIΔ58 obtained from a static analysis of the SAXS data. It must be stressed that the model is not intended to represent the true 3D structure, for which there may be other possibilities (see Supplementary Note), but rather a possible fold which is consistent with our data and as such can be used as a low resolution shape in the ensemble modelling. Next, we generated a large pool of possible conformers (400.000 copies) using the crystal structure of CI-NTD_80_ (Fig. 1) and the model of the dimerization region (Fig. S7B). The flexible linker and the his-tag at the C-terminus of CI∆58 were constructed using the statistical coil generator Flexible-Meccano^20^,^21^. A total of 40 ensembles of 20 conformers each were selected using ASTEROIDS^22^,^23^,^24^ from different starting pools comprising 10.000 conformers in order to obtain sufficient data for a detailed statistical analysis of the conformational space sampled by CI∆58 (see Fig. S8A, B for details on the optimization of ensemble and pool sizes). Each of the individual ensembles fit well to the experimental scattering curve (Fig. 8A). The distribution of the radius of gyration, *R*_g_, in the initial pool (400.000 conformers) and those of the selected structures (800 conformers) shows that CI∆58 is more compact than the initial pool of structures (Fig. 8B). A more thorough analysis of the distribution of inter-domain distances shows that both the distance between the two NTDs (Fig. 8D) as well as the distance between the dimerization region (CTD_1_) and each of the NTDs (Fig. 8C) are slightly shorter than what is expected from a statistical coil sampling of the linker region. The distribution of the NTDs around the centre of mass of the dimerization region in the selected ensembles shows that the two NTDs do not occupy well-defined positions, but rather sample a large conformational space (Fig. 8E–G).

## Discussion

In order to obtain further structural knowledge of the CI repressor from TP901-1, we have employed here a ‘divide and conquer’ strategy which has aided the study of other phage repressors, and investigated truncated variants of CI by a variety of complementary biophysical techniques. These studies show that the helices α6 and α7 constitute the dimerization domain of CI∆58 (and by inference, full-length CI). Another truncation mutant of TP901-1 CI (CI∆78), where only α7 is missing, was found to be monomeric in SEC, confirming that both helices are needed for dimerization. We can thus conclude that TP901-1 has a helical dimerization domain, which to our knowledge makes it unique among characterized phage repressors (which typically dimerize through domains with predominantly β secondary structure).

We thus suggest that the dimerization region (90–122) is renamed to CI-CTD_1_, while the remaining CI-CTD (123-180) is renamed as CI-CTD_2_. This CI-CTD_2_ contains the last ten residues of CI which are essential for higher oligomerization of CI^10^. The extended CI-NTD crystal structure as well as the NMR relaxation data also precisely identifies residues 81–89 of TP901-1 CI as a flexible linker, while residues 75–80 should be included in the CI-NTD.

The dimerization domain (CI-CTD_1_) has been characterized here by SEC and CD showing that it can fold as a helical structure with a clear thermal denaturation transition at 39.3 °C and dimerize/tetramerize without the presence of additional domains, strengthening the argument that this helical subdomain plays a major role in multimerization of CI. The *ab initio* SAXS shape reconstruction of CI∆58 is fully consistent with this dimerization region forming a compact domain/subdomain of its own.

Thermal unfolding of CI∆58 shows two transitions which we assign to the unfolding of CI-NTD and CI-CTD_1_ occurring at 64.2 °C and 42.7 °C, respectively, since the isolated domains showed T_m_s of 58.1 °C and 39.3 °C.

Comparing the NMR DNA titration data of CI-NTD^12^ and CI∆58 shows that the interacting residues are the same and that only residues in the CI-NTD interact with DNA (Fig. S3).

The results presented here in addition show that TP901-1 CI∆58 binds to DNA canonically, with a dimer binding to a single O_L_ operator site. Strong cooperative binding to two adjacent half-sites is evidenced by a combination of native MS and EMSA, with a much stronger interaction of CI∆58 compared to the monomeric CI-NTD.

The ensemble selections obtained here on the basis of the experimental SAXS data show that the CI-NTDs sample a large conformational space prior to DNA binding. Simultaneous interaction between three operator sites (O_R_, O_L_ and O_D_) and the hexameric full-length CI would require some degree of flexibility. Thus, the large conformational space sampled by the NTDs allows CI to ‘search’ for the other operator sites once bound to a given site. This notion is supported by our NMR studies, which demonstrate that upon interaction with DNA, the linker maintains its flexibility.

The linker region is important for the function of the genetic switch. If the linker is elongated by five residues, at position 78 or 89, bi-stability is lost in the presence of functional *mor*^11^ the anti-repressor gene. CI∆58, similarly to the insertion mutants, does not repress in the presence of *mor*^10^, although it binds DNA with an affinity similar to full-length CI. Both in the case of the insertion mutant and CI∆58, the reason for the loss of repressor function might be an increased affinity for MOR, due to increased conformational flexibility and therefore a more accessible MOR binding site. In contrast, a truncation mutant containing 15 additional residues, CI∆43, both binds and represses in the presence of *mor*, and is able to maintain bi-stability *in vivo*. The additional residues could either restrict the flexibility or restrict by other means the accessibility to the MOR binding site. The MOR binding has been suggested^11^,^12^ to take place in a region including part of the scaffolding helix (α2), and a region including the last two helices of NTD. Recent sequence searches (not shown) indicated that α5 and the structured residues immediately following are highly conserved in CI homologues from organisms also containing MOR homologues. Furthermore analysis with the meta-PPISP server (not shown) identified a similar region in TP901-1 CI as a putative protein-protein interaction region. Given its spatial proximity, it seems plausible that one of the functions of the flexible linker could be to regulate access to the MOR binding site.

In this study we have employed complementary techniques to study the structure and dynamics of the CI repressor from TP901-1. We precisely identify the location of the flexible linker, characterize the helical CTD_1_ dimerization domain, and investigate the dynamics of the dimeric CI∆58 truncation construct. Furthermore, we firmly establish that CI∆58 binds canonically and cooperatively to DNA. We propose that the linker region provides full-length CI with the flexibility required to bind three operator sites simultaneously, and that it regulates the access of the antirepressor MOR to its binding site on CI.

## Methods

### Production and basic characterization of CI∆58, CI∆43, CI∆78, CI-NTD, and CTD_1_

The expression and purification of CI-NTD, CI∆58 and CI∆43 truncated versions of CI, containing 74, 122 and 137 residues of CI from TP901-1 respectively, have been described previously^10^,^12^. In addition to CI-NTD, CI∆58 and CI∆43 a new variant denoted CI∆78 was cloned into pQE-70. CI∆78 contains the first 102 residues of CI, also followed by the same eight additional residues (RSHHHHHH) as the other constructs of CI, however in CI∆78 the second amino acid is the native Leu as in CI-NTD.

All constructs were expressed in *E.coli* M15 cells and purified as described previously for CI-NTD^12^. For labelled protein (^15^N or ^15^N, ^13^C CI∆58) minimal M9 medium was used containing ^15^N ammonium chloride and/or ^13^C-glucose (Sigma-Aldrich) as isotope sources. After affinity purification, proteins were further purified by SEC using a HiLoad 26/60 Superdex75 and concentrated using Amicon Ultra Centrifugal filters with a molecular weight cut-off of 10 or 3 kDa. SDS-PAGE, Matrix Assisted Laser Desorption/Ionization- Time of flight (MALDI-TOF) and Electron Spray (ESI) -TOF mass spectrometry (MS) were used to verify purity. A HiLoad 26/60 Superdex75 was used for native molecular weight estimation by comparison with standards Conalbumin (75 kDa), Ovalbumin (43 kDa), Carbonic Anhydrase (29 kDa), Ribonuclease A (13.7 kDa), CI-NTD (9.6 kDa) and Aprotinin (6.5 kDa) (Fig. 2). CI-NTD was included to get better estimation in the low M_w_ range. As the curve deviated from linearity for the highest and lowest M_w_ standards, we also calculated native M_w_ based on standard curves where one or both of these standards were omitted and results are presented in Table S1.

The protein concentration was estimated from the UV absorbance at 280 nm using theoretical extinction coefficients of 8480 M^−1^cm^−1^ for CI∆58 and CI∆78, and 9970 M^−1^cm^−1^ for CI∆43, calculated using the ExPASy tool ProtParam^25^. CTD_1_ was purchased from CASLO as a 35 residues peptide (90-ENIEETITVMKKLEEPRQKVVLDTAKIQLKEQDEQ-125) including three additional residues compared to the minimal helical region identified, to ensure stability. It was delivered as a lyophilized chloride salt with a purity of 98.45%. Initially problems were encountered in solubilizing CTD_1_ in 20 mM NaF buffer and 20 mM Tris-HCl, 100 mM NaCl buffer, both with a pH of 7.5. Raising the pH to 12 with 5 M NaOH when solubilizing the lyophilized chloride salt and subsequently lowering the pH to 7.5 with 85% ortho-phosphoric acid achieved better results with no visible sign of aggregation. Since CTD_1_ does not contain any aromatic residues, protein concentration was estimated by the bicinchoninic acid^26^ (BCA) protein assay using Lysozyme as standard protein.

### Circular Dichroism (CD) spectroscopy measurements of CI∆58 and CI-NTD

Thermal unfolding traces of CD were recorded on a JASCO J-815 instrument at a fixed UV wavelength (222 nm) in a quartz cell with path length 1.0 mm from Hellma. Proteins used for thermal unfolding CD measurements were in 20 mM Tris-HCl, 100 mM NaCl at pH 7.5. CD temperature scans were performed between 293.15 K and 363.15 K, with a ramp rate of 0.5 °C/min to achieve complete thermal denaturation. In case of CTD_1_ the temperature range was set from 273.15 K to 363.15 K, due to early changes in CD signal. The thermal denaturation experiments were carried out at concentrations of 38.9 μM, 25.9 μM and 33.7 μM for CI∆58, CI-NTD and CTD_1_, respectively. By solubilizing CTD_1_ in 20 mM NaF and using a circular quartz cell with path length 0.0050 mm from Hellma, it was possible to record the CD spectrum of CTD_1_ in the range of 260 nm to 178 nm. Concentration of CTD_1_ was determined by BCA to 337 μM.

### NMR spectroscopy studies of CIΔ58

Either ^15^N, ^13^C- or ^15^N-labeled recombinant CIΔ58 was concentrated in NMR buffer (20 mM Tris, 100 mM NaCl, pH 6.5, 10% v/v D_2_O) to 395 μM or 948 μM. The NMR spectra of CIΔ58 were assigned using triple resonance experiments (HNCO, HN(CA)CO, HN(CO)CA, HNCA) acquired at 311 K and a magnetic field strength of 600 MHz. All recorded data were transformed using NMRPipe^27^ and analysed in CcpNmr^28^ or SPARKY (Goddard and Kneller, unpublished). The spectra were peak picked using a macro included in CcpNmr followed by manual verification. Sequential connectivities were obtained using Nexus under CcpNmr or by MARS^29^. An almost complete assignment of the ^1^H^N^, ^15^N, ^13^Cα and ^13^C’ nuclei was obtained (BMRB ID: 26058). Secondary chemical shifts were calculated using random coil values from refDB^30^.

^15^N *R*_1_, *R*_2_ (CPMG) relaxation experiments were carried out at 298 K on a Varian spectrometer operating at a ^1^H frequency of 600 MHz^31^ using a ^15^N, ^13^C-labeled sample of CI∆58 (395 μM) and a ^15^N, ^13^C-labeled sample of CI-NTD (700 μM). In the case of CI∆58, the assignment of the ^1^H-^15^N HSQC spectrum at 311 K was transferred to the spectrum recorded at 298 K using a series of HSQC spectra recorded in this temperature range. The CI-NTD spectrum was assigned previously^12^. The peak intensities at each delay for *R*_1_ relaxation (0, 100, 200, 400, 600, 800, 1100, 1500 and 1900 ms), and *R*_2_ relaxation (0, 30, 50, 70, 90, 130, 170, 210 and 250 ms) were fitted to an exponential decay. To obtain estimates of errors on the relaxation rates, a repeat measurement of one of the relaxation delays was performed in each case (600 ms for *R*_1_ and 70 ms for *R*_2_). The analysis of relaxation data in terms of overall correlation times was done using Tensor^32^.

### DNA interaction with CI∆58 studied with NMR spectroscopy, native MS, and EMSA

Previously, NMR studies of the DNA:CI interaction were carried out with CI-NTD and the O_L_ site^12^. Similar titration experiments were carried out with the dimeric variant (CI∆58) and an 18 bp oligonucleotide representing O_L_ (5′-GTTCATGAAACGTGAACT-3′). Increasing amounts of DNA were added to the protein and for each step of the titration a ^1^H-^15^N HSQC spectrum was recorded at a ^1^H frequency of 800 MHz and 298 K. The titration series of CI∆58 and DNA was carried out at a sodium chloride concentration of 300 mM leading to shifting and disappearing CI∆58 resonances according to a fast to intermediate exchange regime. HSQC spectra were recorded for the following concentrations of CI∆58: O_L_; (113.6 µM:0.0 µM), (110.3 µM:12.2 µM), (108.7 µM:21.5 µM), (106.7 µM:30.5 µM), (103.6 µM:41.0 µM), (101.2 µM:51.2 µM), (98.4 µM:62.8 µM) and (90.0 µM:97.0 µM) reaching a 1:1.08 concentration ratio of dimer to double stranded DNA at the final titration point.

CI∆58, CI-NTD and their DNA complexes were analysed by native MS^33^ in 250 mM ammonium acetate (99% pure) buffer at pH 6.8. The following concentrations were used: apo CI∆58 (6 μM), CI∆58:O_L_ (4.3 μM:2.1 μM) and CI-NTD:O_L_ (2.2 μM:1.3 μM). Protein ions were generated using a nanoflow electrospray (nano-ESI) source. Nanoflow platinum-coated capillaries were bought from Thermo Electron SAS (Courtaboeuf, France). MS analyses were carried out on a quadrupole time-of-flight mass spectrometer (Ultima, Waters Corporation, Manchester, U.K.). The instrument was modified for the detection of high masses^34^,^35^. All mass spectra were calibrated externally using a solution of cesium iodide (6 mg/mL in 50% isopropanol) and were processed with the Masslynx 4.0 software (Waters Corporation, Manchester, U.K.) and with Massign software package^36^.

EMSA experiments were performed essentially as previously described^3^, except for the use of Cy5-labeling instead of radioactive labeling and agarose gel electrophoresis instead of polyacrylamide gel electrophoresis. In each binding assay, approximately 0.5 nM primer was mixed with varying concentrations of CI∆58 or CI-NTD in a total of 20 μl binding solution^3^. After pre-incubation on ice without DNA for 15 min, the DNA fragment was added and incubation was continued for 30 min. The mixture was then pipetted into the empty wells of a chilled 2% agarose gel and run horizontally in 1x TBE (Tris/Borate/EDTA) buffer (Thermo Scientific) for 90 min at 10 V/cm at 0 °C. For visualization and quantification the gel was scanned directly in a STORM 860 Imager (Amersham Biosciences) using the red channel (635 nm) at high sensitivity, followed by analysis using the ImageQuant TL software (Amersham Biosciences).

### Crystallographic studies of extended NTD

Attempts were made to co-crystallize CI∆43 (18.6 mg/ml or 1.1 mM) and a 19bp DNA ds oligonucleotide including the O_L_ sequence 5′-AGTTCATGAAACGTGAACT-3′ (0.5 mM) in an approximately 1:1 binding ratio under the assumption that CI∆43 binds the DNA fragment as a dimer. After more than a year a cluster of intergrown plates appeared using 0.01 M magnesium chloride hexahydrate, 0.05 M HEPES sodium pH 7.0, 2.0 M ammonium sulfate as precipitant in a hanging drop originally set up with 1 μl of each of protein-DNA complex, reservoir, and milli-Q H_2_O.

A data set was collected P11, PETRA III, DESY, Hamburg, Germany using a PILATUS detector and exploiting the microfocus (1 μm) capabilities of the beamline, in order to collect diffraction primarily from a single plate. Only selected images were used for processing the final data set in XDS/XSCALE^37^ in order to minimize the problem of diffraction from multiple plates. This resulted in almost complete data to 2.1 Å resolution (see Table S2) with the space group P2_1_2_1_2 and with cell dimensions of a = 53.72 Å, b = 36.01 Å and c = 38.77 Å.

Molecular Replacement (MR) was carried out in PHENIX^38^ using the structure of NTDQ45A/N48A-CI as a model (PDB 3ZHI). It was clear from the MR solution that no DNA was present in the crystal, where furthermore no amino acid residues were visible beyond residue 80. The MR result yielded a rotational function Z-score of 5.8, a top translation function Z-score of 8.2, and a log-likelihood (LLG) of 387.942. The initial isotropically restrained refinement of the placed model lowered the R_work_/R_free_ from 40.86%/38.86% to R_work_/R_free_ of 29.68%/38.76%. Eventually the final structure was refined to R_work_/R_free_ of 21.58%/23.59% with good geometry. Data and model statistics are summarized in Table S2.

### Small Angle X-ray Scattering (SAXS) data collection, processing and modelling

SAXS data of CI∆58 were collected at station ID14-3 at the European Synchrotron Radiation Facility Grenoble, France using a PILATUS detector. Data were collected using a sample to detector distance of 2.43 m and a temperature of 298 K covering a range of momentum transfer of 0.05 < *s* < 5 nm^−1^, where *s* = 4πsinθ/λ, 2θ is the scattering angle and λ = 0.931 Å.

Protein concentrations of 2.6, 4.2 and 9.6 mg/ml in 1 M NaCl were used, while a concentration of 4.02 mg/ml was used for Bovine Serum Albumin (BSA). Data processing steps as background subtraction, scaling and merging were performed using the program PRIMUS^39^. The forward scatter, *I*(0), and the radius of gyration, *R*_g_, were obtained using the Guinier approximation^40^ or from the entire scattering profile using AUTOGNOM^41^. AUTOGNOM also provides the pair distribution function of the particle, P(*r*), and the maximum particle dimension, D_max_. The molecular mass (M_w_) was estimated from the Guinier approximation and was crosschecked by calculating the excluded volume (V_p_) of the hydrated particle from the Porod equation^42^ in AUTOPOROD^41^.

Ten *ab initio* models were calculated using GASBOR22i^43^ with default settings except when P2 symmetry was imposed. The reconstructed models were aligned with SUPCOMB20^44^ and an average *ab initio* model was calculated by DAMAVER^45^. DAMFILT was used to represent the average *ab initio* model as a more compact and probable model by removing low occupancy and loosely connected dummy residues.

Structural models of CI∆58 were obtained using the crystal structure of CI-NTD (residues 1–80) and the sequence homology model of the dimerization region. A total of 400.000 structures were generated, where the flexible linker and the his-tag at the C-terminus were constructed using Flexible-Meccano^20^,^21^. Side chains were added to each structure using SCCOMP^46^ and SAXS curves were calculated using CRYSOL^47^. Sub-ensembles of CI∆58 were selected using ASTEROIDS^22^,^23^,^24^ on the basis of the experimental SAXS data as described in the Results section.

### Sequence and structure analysis

Fold recognition searches using the sequence of CI∆58 were carried out using the Protein Homology/analogY Recognition Engine V 2.0 (PHYRE2)^48^,^49^. The structure of the extended CI-NTD was submitted to the meta.PPISP server^50^ to detect regions potentially involved in protein-protein interactions.

## Additional Information

**How to cite this article**: Rasmussen, K. K. *et al.* Structural and dynamics studies of a truncated variant of CI repressor from bacteriophage TP901-1. *Sci. Rep.* **6**, 29574; doi: 10.1038/srep29574 (2016).

## Supplementary Material

Supplementary Information
