# Supplementary material for: Structural and dynamics studies of a truncated variant of CI repressor from bacteriophage TP901-1
Source: Sci Rep. 2016 Jul 12;6:29574. doi: 10.1038/srep29574 (PMC4941734; doi:10.1038/srep29574)
Supplement: Supplementary Information [file srep29574-s1.pdf]

Supplementary Information

Structural and dynamics studies of a truncated variant of CI repressor from bacteriophage TP901-1

Kim Krighaar Rasmussen[1], Kristian E. H. Frandsen[1], Elisabetta Boeri Erba[2], Margit Pedersen[3], Anders K.Varming[1], Karin Hammer[4], Mogens Kilstrup[4], Peter W. Thulstrup[1], Martin Blackledge[2], Malene Ringkjøbing Jensen[2,*] and Leila Lo Leggio[1,*]

[1]Department of Chemistry, University of Copenhagen, Universitetsparken 5, Copenhagen, Denmark
[2]Univ. Grenoble Alpes, CNRS, CEA, Institut de Biologie Structurale, Grenoble, France
[3] Department of Biology, University of Copenhagen, Ole Maaløes vej 5, DK-2200 Copenhagen N, Denmark
[4] Metabolic signalling and regulation, Department of Systems Biology, Technical University of Denmark, DK-2800 Lyngby, Denmark.

Running title: Dynamics of CI Repressor from TP901-1

Keywords: phage repressor; genetic switch; helix-turn-helix transcription factor; dimerization domain; ensemble analysis

[1] To whom correspondence should be addressed

Dr. Leila Lo Leggio
E-mail: leila@chem.ku.dk
Tel: +45 35 32 02 95

Dr. Malene Ringkjøbing Jensen
E-mail: malene.ringkjobing-jensen@ibs.fr
Tel: +33 4 57 42 86 68

Table S1. Calculated M_w from Size exclusion chromatography

Protein	M_{wExp} (Da)*	Ratio M_{wExp}/M_w*	M_{wExp} (Da)**	Ratio M_{wExp}/M_w**	M_{wExp} (Da)***	Ratio M_{wExp}/M_w***	M_{wExp} (Da)****	Ratio M_{wExp}/M_w****	M_w (Da)	Estimated oligomeric state #
**CIΔ43**	39741	2.35	33663.4	1.99	41546.7	2.45836	43926.5	2.60	16900.2	2 (3)
**CIΔ58**	33483.3	2.22	27463.9	1.82	34463.9	2.28947	36474.0	2.42	15053.2	2
**CIΔ78**	17897.8	1.41	13034.4	1.02	17383.7	1.36842	18464.1	1.45	12703.5	1
**CI-CTD$_1$ peak1**	18170.3	4.37	13286.3	3.2	17691.9	4.25552	18789.8	4.52	4154.7	4 (3 or 5)
**CI-CTD$_1$ peak2**	11766.5	2.83	7929.12	1.90	11013.2	2.64906	11725.9	2.82	4154.7	3 (2)
**CI-CTD$_1$ peak3**	4778.57	1.15	2718.49	0.65	4121.03	0.991252	4410.5	1.06	4154.7	1

* Masses determined from standard curve calculated from mentioned standards excl. Conalbumin 75 kDa

** Masses determined from standard curve calculated from mentioned standards excl. Aprotinin 6.5 kDA

*** Masses determined from standard curve calculated from mentioned standards excl. Conalbumin and Aprotinin (75 kDa, and 6.5 kDa)

**** Masses determined from standard curve including all mentioned standards

From closest integer to MwExp/Mw (if more than one possibility, alternatives are given in brackets)

Table S2. Data collection and refinement statistics

	NTD80
**Data collection**	
Wavelength (Å)	1.033
Space group	$P2_12_12$
Cell dimensions	
a, *b*, *c* (Å)	53.72 36.01 38.77
(°)	90.00, 90.00, 90.00
Resolution (Å)	30.00-2.10 (2.16-2.10)
R_{meas}	17.2 %(81.3 %)
I / s*I*	5.68 (1.73)
Completeness (%)	90.6 (87.3)
Redundancy	3.52 (2.89)
**Refinement**	
R_{work} / R_{free}	21.58 % / 23.59 %
R.m.s. deviations	
Bond lengths (Å)	0.004
Bond angles (°)	0.681
Ramachandran plot	
Favoured	100 %
Outliers	0 %

Table S3. Analysis of experimental SAXS data of CIΔ58 and bovine serum albumin (BSA)

	R_g [a] (nm)	R_g [b] (nm)	V_p [c] (nm^3)	M_{wExp} [d] (kDa)	M_{wExp} [e] (kDa)	M_w [f] (kDa)
BSA	3.09	3.14	114.58	-	71.61	66.46
CIΔ58	3.12	3.10	55.66	31.79	34.79	30.11

[a]Radius of gyration, R_g, estimated from the Guinier approximation.
[b]R_g estimated by AutoGNOM.
[c]The excluded hydrated volume (Porod volume), V_p.
[d]Molecular weight, M_w, estimated from $I(0)$ obtained by the Guinier approximation.
[e]M_w estimated from AutoGNOM.
[f]Theoretical M_w.

Table S4. Analysis of experimental SAXS data. Weighted mean χ^2 calculated from single reconstruction models and the normalized spatial discrepancy (NSD) calculated by DAMAVER when aligning the ten reconstructed models.

Ab initio model	χ^2 weighted mean	NSD mean
CIΔ58 P1	1.053 ± 0.036	1.038 ± 0.012
CIΔ58 P2	1.153 ± 0.099	1.132 ± 0.083

Table S5. Top six hits from a PHYRE2 search carried out on the primary sequence of CIΔ58 (residues 1-122 of full-length CI).

	PDB	Alignment coverage (%)[a]	Confidence	% Identity	Template
1	3OP9	98 (1-121)	99.8	15	Transcriptional regulator from *Listeria innocua* (tetramer)
2	3IVP	97 (3-122)	99.8	15	The structure of a possible transposon-related DNA-binding protein from *Clostridium difficile* 630 (dimer)
3	3LIS	90 (5-115)	99.8	22	Crystal structure of the restriction-modification controller protein C.Csp231I (dimer)
4	2KPJ	70 (1-87)	99.7	21	Solution structure of protein SOS-response transcrip-tional repressor, LexA From Eubacterium rectale (monomer)
5	1B0N	94 (5-120)	99.7	19	SinR/SinI protein complex (heterodimer)
6	3QYX	95 (5-122)	99.7	15	Crystal structure of Mycobacterium tuberculosis EspR in complex with a small DNA fragment (tetramer)

[a]Numbers in brackets refer to the region of the protein that has been aligned.

Figure S1.

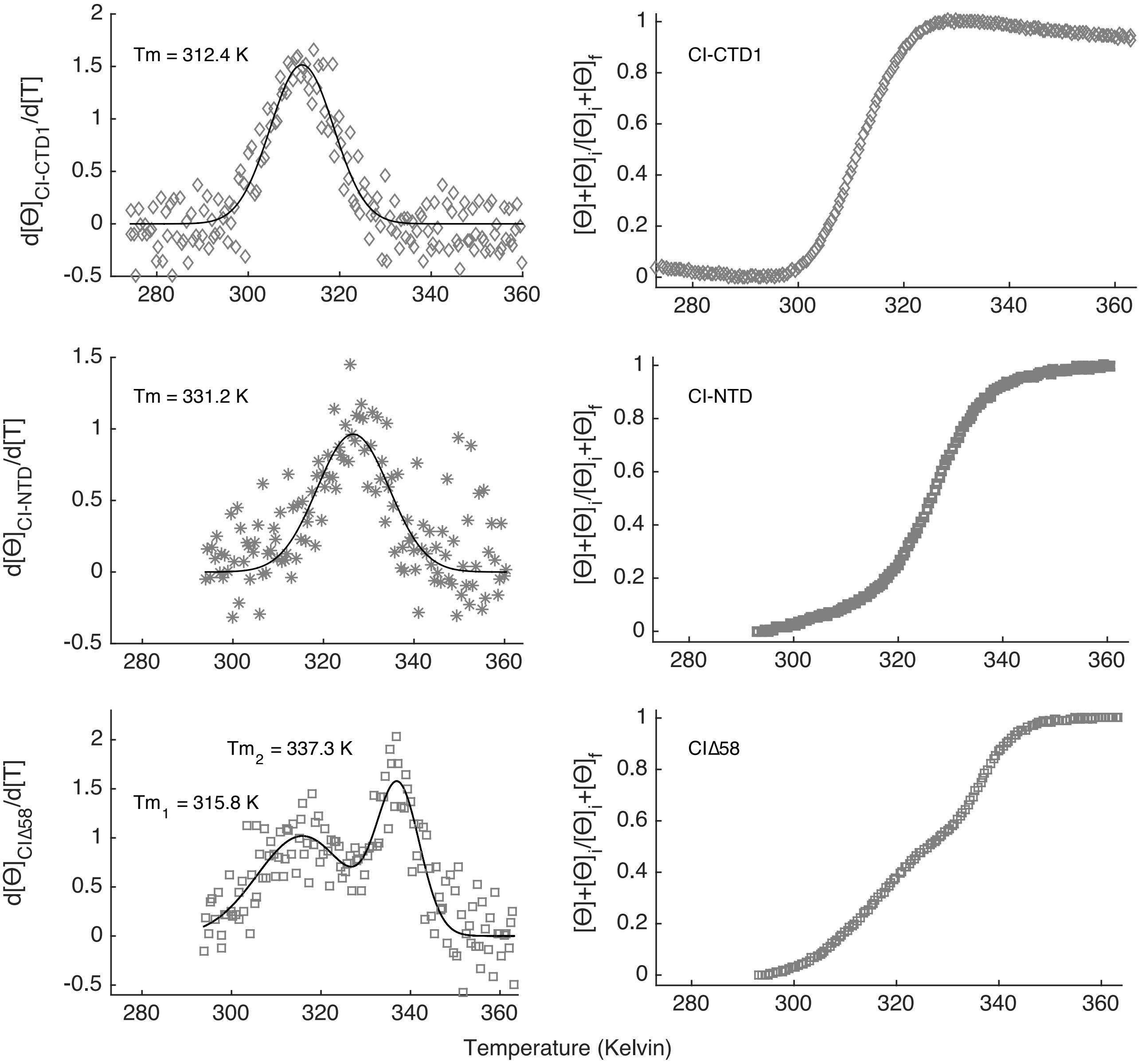

Figure S1. Thermal unfolding of CIΔ58,CI-NTD and CTD₁. By measuring the change in ellipticity (θ) with temperature at 222 nm it was possible to identify a three state unfolding event for CIΔ58, which is clear when comparing the unfolding profile to that of CI-CTD1 (diamond) and CI-NTD (asterisk, residues 1-74). The melting temperature (T_m) was determined by taking the first derivative ($d[\theta]_{protein}/dT$) (full line). For CI-CTD1 and CI-NTD T_m = 312.4 K and T_m = 331.2 K (39.3 and 58.1 °C) were obtained, respectively. Whereas for CIΔ58 (square) the two melting temperatures were determined to be T_{m1} = 315.8 K (42.7 °C) and T_{m2} = 337.3 K (64.2 °C). A straightforward interpretation is that the dimerization region is less stable compared to NTD, and unfolds first. The increased thermal stability of NTD in the context of CIΔ58 maybe due to the additional residues (residues 74-80) that in the crystal structure interact closely with the rest of the domain. This clearly shows that CIΔ58 contains two domains, in good agreement with SAXS results.

Figure S2.

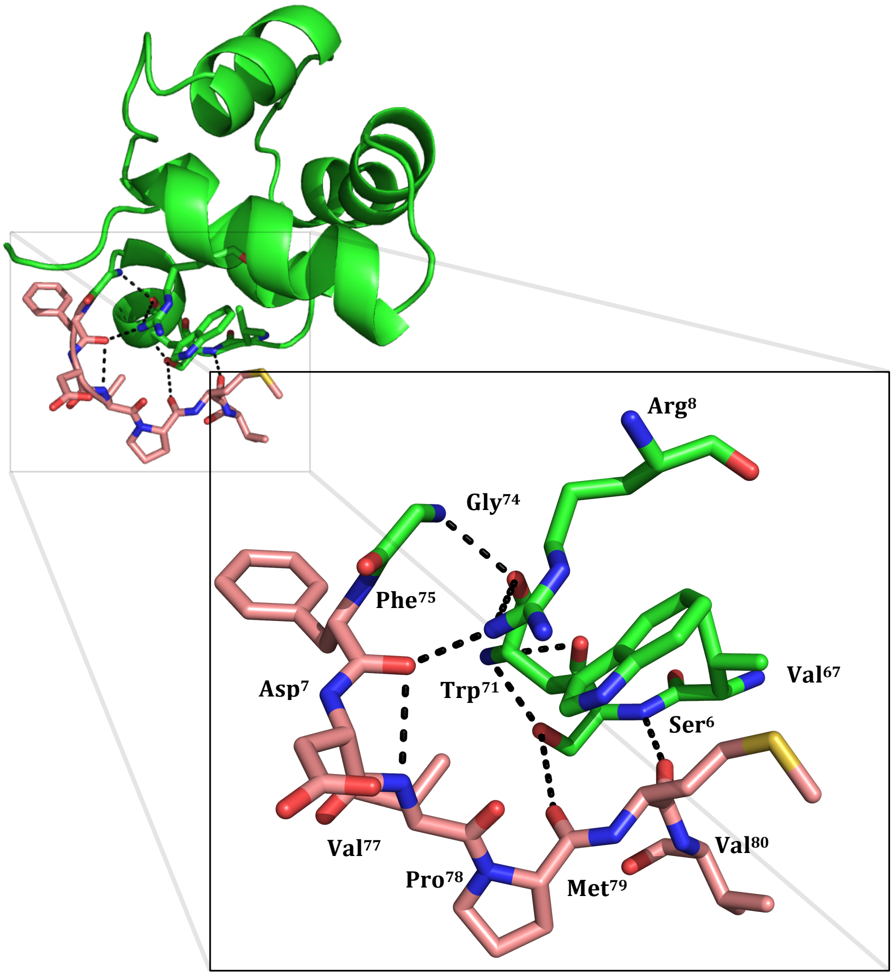

Figure S2. Crystal structure of CI-NTD80 showing the network of hydrogen bonds stabilizing the unstructured part of CI-NTD80. In the background is full structure of CI-NTD80 showing the location of the extra six residues (salmon) CI-NTD80, and in foreground a close up on the residues involved in stabilizing the local structure of CI-NTD80. Hydrogen bonds are shown in dashed lines (black).

Figure S3.

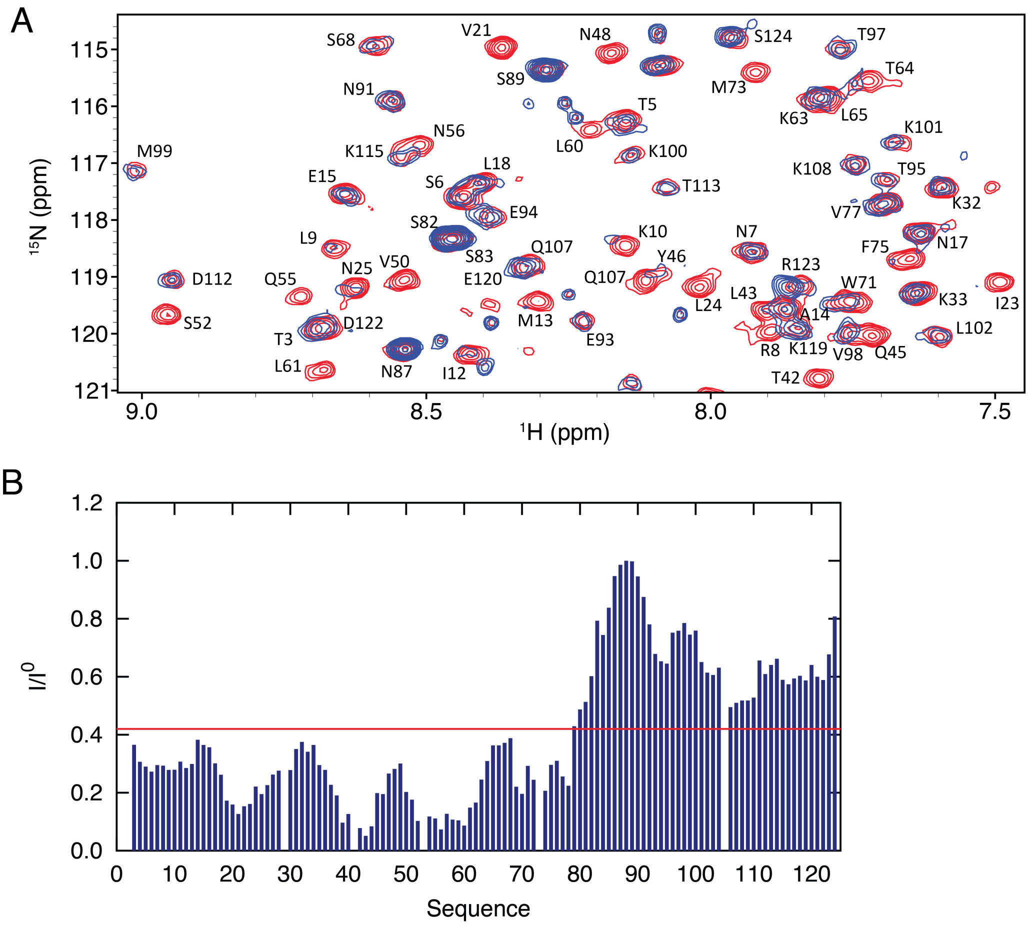

Figure S3. Mapping the interaction site between dimeric CIΔ58 and the full-length operator site (O_L). (A) Superposition of HSQC spectra of CIΔ58 in the absence (red) and presence of a stoichiometric amount of DNA (blue). (B) Ratio of intensities in the HSQC spectra of CIΔ58:DNA. I^0 corresponds to CIΔ58 (113.6, 0.0 μM) alone and *I* corresponds to CIΔ58:DNA (90.0:97.0 μM*)*. The intensity ratios were smoothed over five residues. The red line is the weighted mean of the intensity ratios along the sequence of CIΔ58. The errors on the residue-specific intensity ratios were obtained using noise estimates in the two HSQC spectra recorded in the absence and presence of DNA. Residues with intensities ratios below the red line were considered to be significant and identify the NTD as the DNA binding domain.

Figure S4.

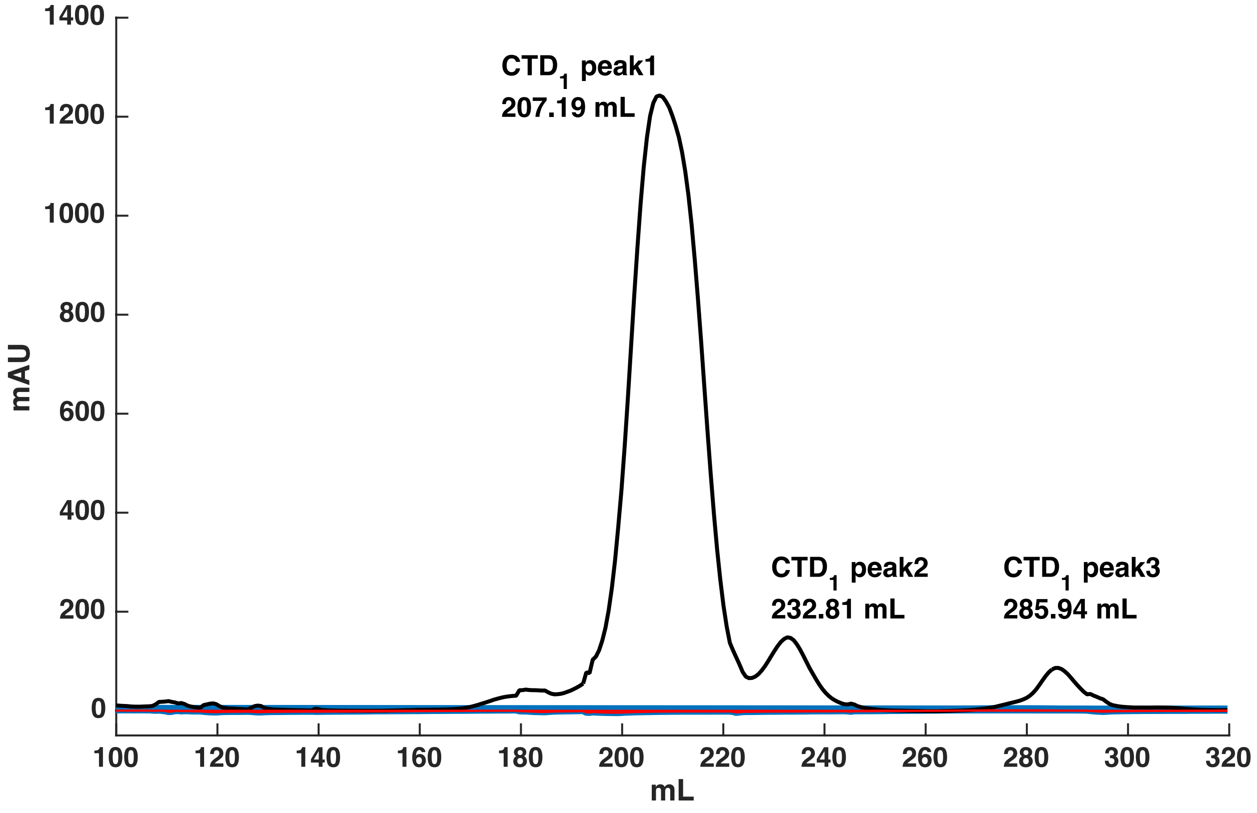

Figure S4. Elution profile of CTD_1 obtained by size exclusion chromatography (SEC). CTD_1 was solubilized and loaded onto a calibrated SuperDex75 Prepgrad. The CTD_1 peptide itself is not detectable by A_{280} nm (blue) or A_{260} nm (red), as it does not contain any Tryptophan or Tyrosine residues, hence we detect elution by measuring A_{214} nm (black).

Figure S5.

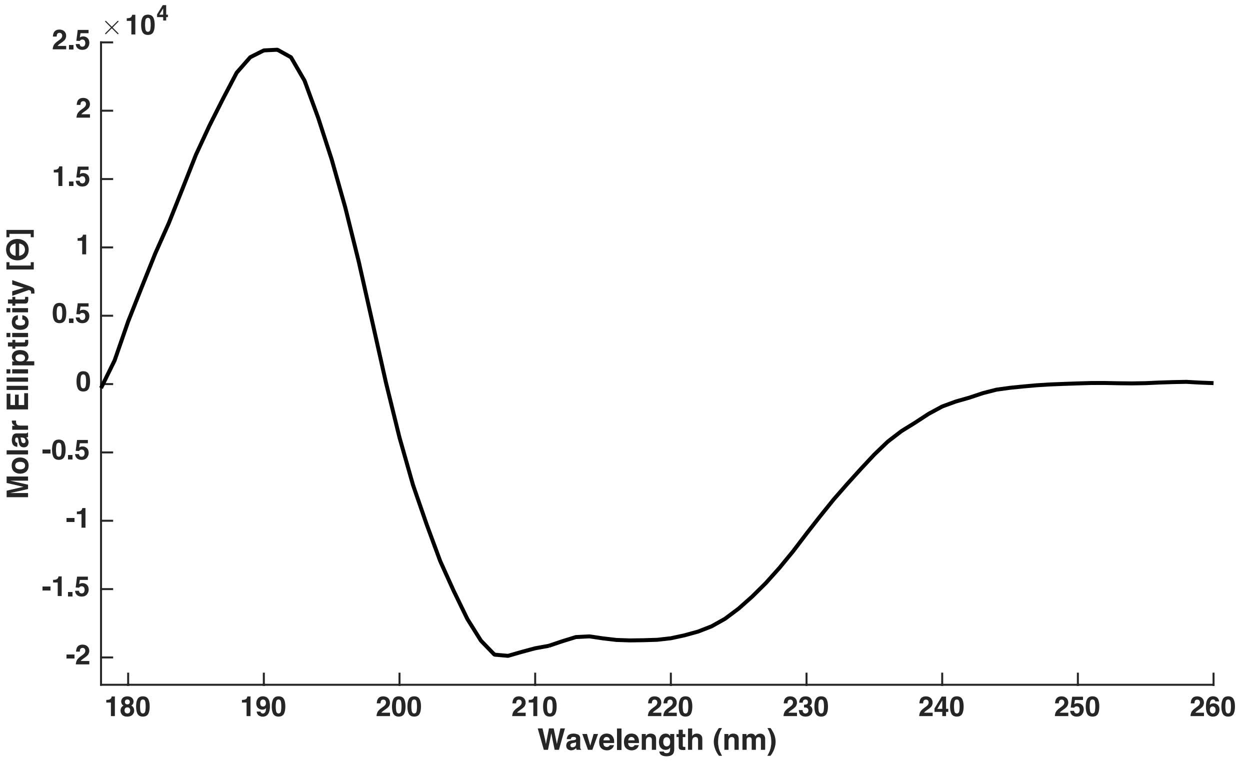

Figure S5. Circular Dichroism measurements of CTD1 display an α-helical spectrum. From the recorded CD spectrum, α-helix content was estimated to 60% by using DichroWeb with CDSSTR reference set 1, 2 and 5.[1,2]

Figure S6.

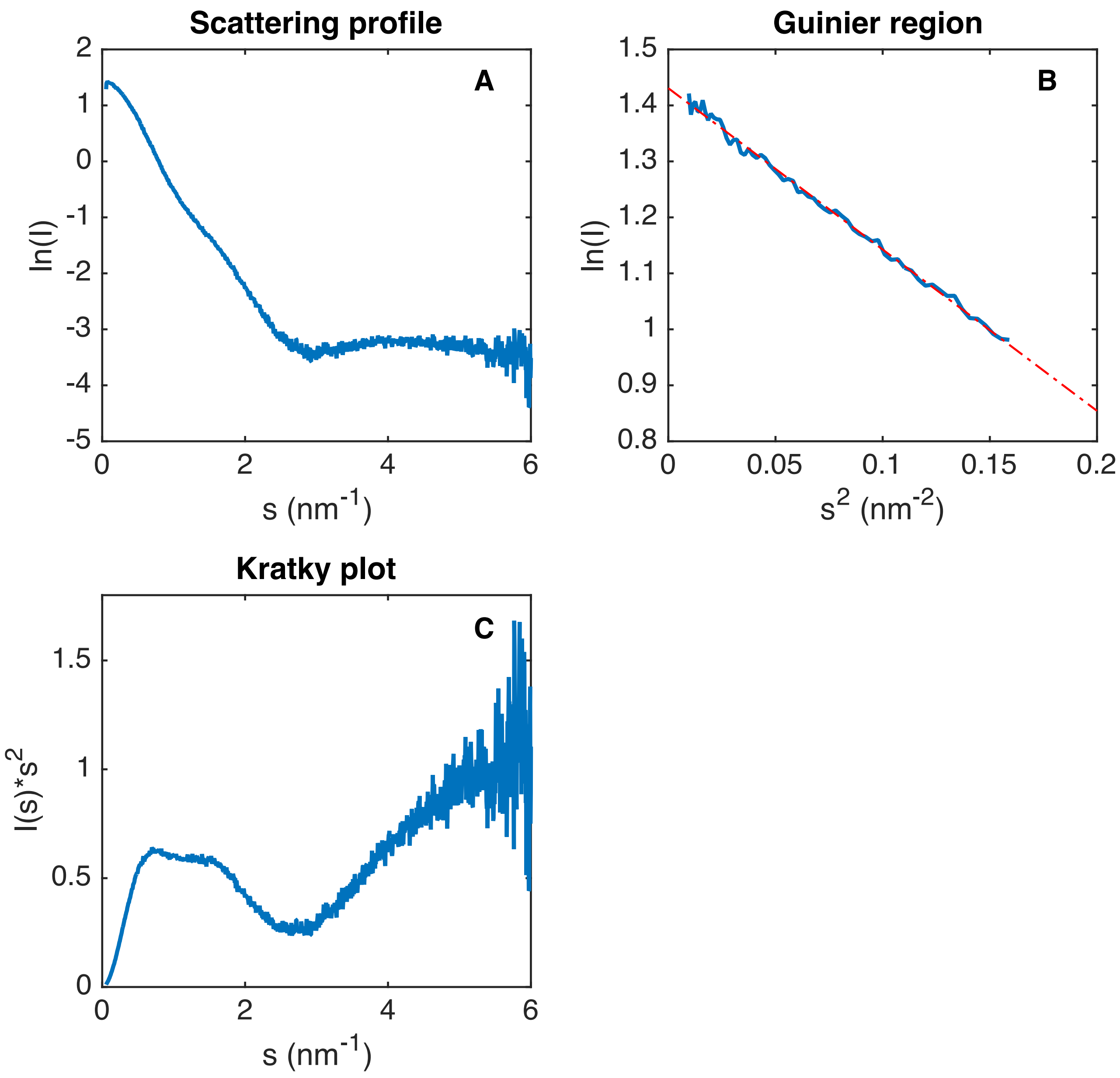

Figure S6. Analysis of SAXS data. A) Background subtracted scattering profile of CIΔ58 plotted as ln(I) versus s. B) Guinier region of the scattering data plotted as ln(I) versus s^2. I(0) was estimated with a linear fit (— · —) to Guinier region. C) Kratky plot of CIΔ58 plotted as I(s)*s^2, showing that CIΔ58 contains both folded and flexible domains.

Figure S7.

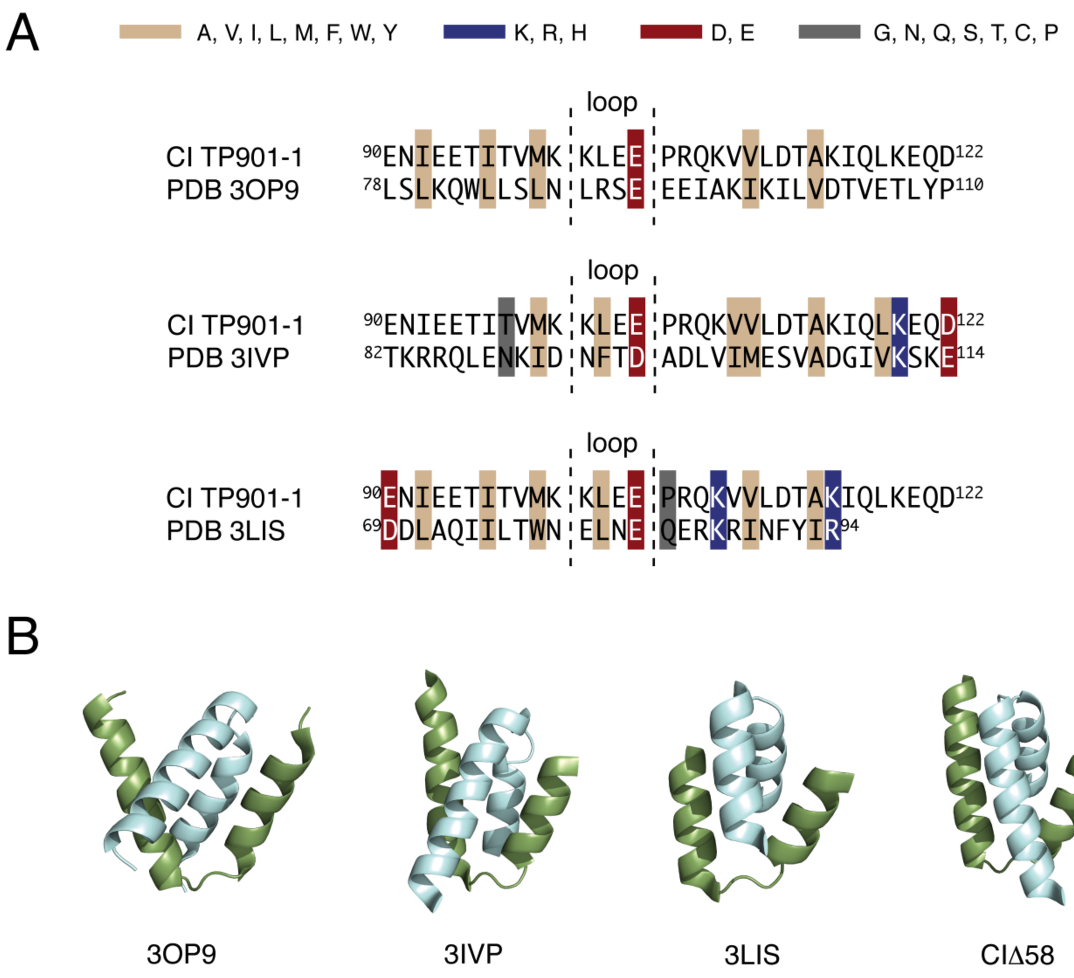

Figure S7. Structural model of the dimerization region of CIΔ58. (A) Alignment of the sequence of the dimerization region of CIΔ58 from TP901-1 with the sequences of the transcriptional regulator from *Listeria innocua* (PDB 3OP9), of the possible transposon-related DNA-binding protein from *Clostridium difficile* 630 (PDB 3IVP) and of the restriction-modification controller protein C.Csp231I (PDB 3LIS). The three proteins were identified as top hits in a PHYRE2 search targeting the primary sequence of CIΔ58. The sequences are colour coded according to conservation of hydrophobic (beige), negatively charged (red), positively charged (blue) and other residues (gray). (B) Structures of the dimerization domains of the three proteins described under (A) as well as the proposed model of the dimerization region of CIΔ58.

Figure S8.

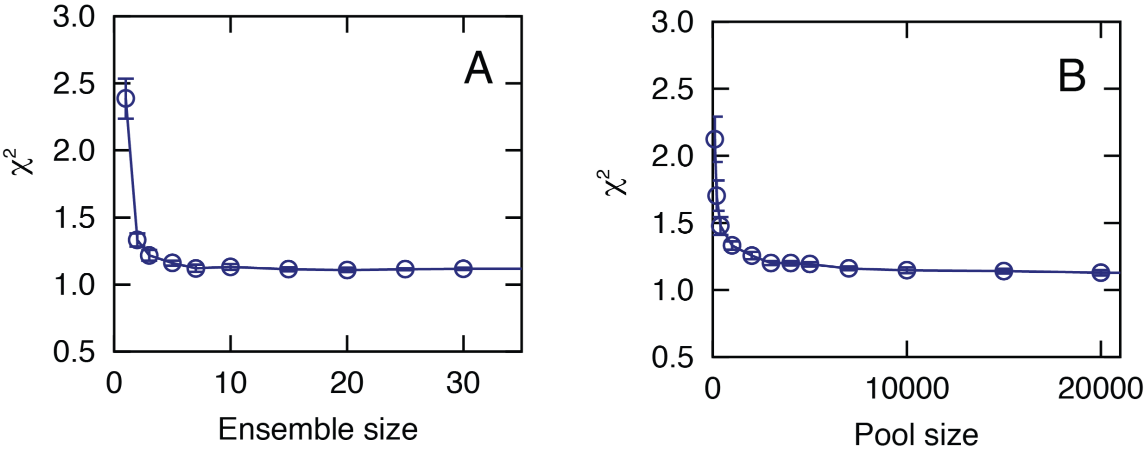

Figure S8. (A) Determination of the optimal ensemble size for describing the conformational dynamics of CIΔ58. The χ^2 is reported between the experimental SAXS curve and that back-calculated from selected ASTEROIDS ensembles of different sizes. Error bars indicate standard deviations obtained by eight repeated selections from different starting pools each comprising 50000 conformers. (B) Determination of the minimum pool size. The χ^2 is reported between the experimental SAXS curve and that back-calculated from selected ASTEROIDS ensembles comprising 20 conformers each. The size of the pool from which the ensembles were selected was varied.

Supplementary note

Attempts to model the dimerization region

In order to obtain a structural model of the dimerization region, the sequences of CTD_1 and CIΔ58 was run through PHYRE2[3,4], a fold recognition server. Searches with the CTD_1 sequence alone returned models with a maximum of 51% sequence coverage, which was deemed too little. Searches with CIΔ58 returned a number of high confidence hits for which the predicted secondary structure is in close agreement with the NMR data (Fig. 2B), including the two α-helices forming the dimerization region (residues 90-101 and 104-120). The top six hits (Table S5), all have similar helical hooks dimeric structure and for which the server gives over 99.5% confidence, but very low sequence identity.

The CI-CTD_1 sequence was also submitted to the *ab initio* prediction QUARK server[5]. The top 10 models were all helical as expected, four representing essentially a single helix and the remaining being two helices with various degrees of bending between them. One of the models closely matched the helix position as determined experimentally by NMR, and could be envisaged to dimerize as a helical hook, however another possibility consistent with all available data is that the dimerization region folds as a coiled coil of bent helices.

In order to have a molecular model for the CTD_1 for the SAXS ensemble analysis we chose the structure of the two helical hooks of *Cc*RM as a template. As the template is slightly shorter than the sequence of CIΔ58 (Fig. S7A), we extended the second helix of the dimerization region with six residues in helical conformation as observed experimentally from NMR (Fig. 3B). The resulting model of the dimerization region of CIΔ58 has most of its hydrophobic residues in the core of the structure. It must be stressed that the model is not intended to represent the true 3D structure, for which there may be other possibilities, but rather a possible fold which is consistent with the NMR, bioinformatics and static SAXS analysis, and as such can be used as a low resolution shape in the ensemble modelling.

References

1. Whitmore, L. & Wallace, B. A. DICHROWEB, an online server for protein secondary structure analyses from circular dichroism spectroscopic data. *Nucleic Acids Research* **32,** W668–W673 (2004).
2. Sreerama, N. & Woody, R. W. Estimation of protein secondary structure from circular dichroism spectra: comparison of CONTIN, SELCON, and CDSSTR methods with an expanded reference set. *Anal. Biochem.* **287,** 252–260 (2000).
3. Kelley, L. A. & Sternberg, M. J. E. Protein structure prediction on the Web: a case study using the Phyre server. *Nat Protoc* **4,** 363–371 (2009).
4. Jones, D. T. Protein secondary structure prediction based on position-specific scoring matrices. *J. Mol. Biol.* **292,** 195–202 (1999).
5. Xu, D. & Zhang, Y. Ab initio protein structure assembly using continuous structure fragments and optimized knowledge-based force field. *Proteins: Structure, Function, and Bioinformatics* n/a–n/a (2012). doi:10.1002/prot.24065
